# 二代酪氨酸激酶抑制剂一线治疗慢性髓性白血病慢性期患者发生严重血细胞减少的相关因素及其对治疗反应和结局的影响

**DOI:** 10.3760/cma.j.issn.0253-2727.2023.04.006

**Published:** 2023-04

**Authors:** 子郁 李, 亚溱 秦, 悦云 赖, 红霞 石, 悦 侯, 小帅 张, 倩 江

**Affiliations:** 北京大学人民医院、北京大学血液病研究所、国家血液系统疾病临床医学研究中心，北京 100044 Peking University People's Hospital, Peking University Institute of Hematology, National Clinical Research Center for Hematologic Disease, Beijing Key Laboratory of Hematopoietic Stem Cell Transplantation, Beijing 100044, China

**Keywords:** 白血病，髓样，慢性, 酪氨酸激酶抑制剂, 血细胞减少, 治疗结果, Leukemia, myeloid, chronic, Tyrosine kinase inhibitors, Cytopenia, Treatment outcome

## Abstract

**目的:**

探索二代酪氨酸激酶抑制剂（TKI）一线治疗慢性髓性白血病慢性期（CML-CP）患者发生严重血细胞减少的相关因素及其对治疗反应和结局的影响，并建立严重血细胞减少的预测模型。

**方法:**

纳入2008年9月至2021年11月间在北京大学人民医院确诊并服用二代TKI作为一线治疗的成年CML-CP连续病例。采用二元Logistic模型、Fine-gray模型和Cox回归模型进行分析。

**结果:**

共收集267例患者，中位年龄36（18～73）岁，男性156例（58.4％），服用尼洛替尼239例（89.5％），达沙替尼28例（10.5％）。43例（16.1％）患者在一线治疗开始后1.0（0.1～3.0）个月发生≥ 3级中性粒细胞和（或）血小板减少，持续1.0（0.1～24.6）个月。男性（*OR*＝2.9，95％*CI* 1.2～6.8，*P*＝0.018）、初诊年龄≥36岁（*OR*＝3.2，95％*CI* 1.4～7.2, *P*＝0.005）、脾脏肋缘下≥ 7 cm（*OR*＝2.8，95％*CI* 1.2～6.6，*P*＝0.020）以及HGB<100 g/L（*OR*＝2.9，95％*CI* 1.3～6.8，*P*＝0.012）与发生≥ 3级中性粒细胞和（或）血小板减少显著相关。根据回归系数，男性、初诊年龄≥36岁，脾脏肋缘下≥7 cm以及HGB<100 g/L各赋1分，可将患者分为低危、中危和高危3组，各组间血细胞减少发生率差异有统计学意义（*P*<0.001）。持续>2周的严重血液学不良反应与较低的完全细胞遗传学反应（*HR*＝0.5，95％*CI* 0.3～0.7，*P*<0.001）和主要分子学反应（*HR*＝0.4，95％*CI* 0.3～0.8，*P*＝0.004）获得率显著相关，与二代TKI治疗失败、疾病进展和生存期无关。

**结论:**

男性、初诊年龄≥36岁、脾脏肋缘下≥ 7 cm以及HGB<100 g/L与初发CML-CP患者服用二代TKI期间发生严重血细胞减少显著相关，联合四者建立的预测模型有助于识别严重血细胞减少的发生风险。严重血细胞减少持续>2周是细胞遗传学和分子学反应的不利因素。

## Abstract (en)

**Objective:**

To explore the influencing covariates of severe neutrophils and/or thrombocytopenia and their effect on treatment response and outcome in patients with chronic-phase chronic myeloid leukemia（CP-CML）receiving initial second-generation tyrosine kinase inhibitors（2G-TKI）.

**Methods:**

Data from consecutive patients aged ≥18 years with newly diagnosed CP-CML who received initial 2G-TKI at Peking University People's Hospital from September 2008 to November 2021 were interrogated. Binary logistic regression models and Fine-Gray and Cox regression models were applied.

**Results:**

Data from 267 patients who received initial 2G-TKI, including nilotinib（*n*＝239, 89.5％）and dasatinib（*n*＝28, 10.5％）, were interrogated. The median age was 36（range, 18–73）years, and 156（58.4％）patients were male. At a median treatment period of 1.0（0.1–3.0）month, 43（16.1％）patients developed grade ≥3 neutrophils and/or thrombocytopenia and recovered within 1.0（0.1–24.6）month. Male（*OR*＝2.9, 95％*CI* 1.2-6.8; *P*＝0.018）, age of ≥36 years（*OR*＝3.2, 95％ *CI* 1.4–7.2, *P*＝0.005）, a spleen below a costal margin of ≥7 cm（*OR*＝2.8, 95％ *CI* 1.2–6.6, *P*＝0.020）, and a hemoglobin（HGB）level of <100 g/L（*OR*＝2.9, 95％ *CI* 1.3–6.8, *P*＝0.012）at diagnosis were significantly associated with grade ≥ 3 neutrophils and/or thrombocytopenia. Based on their regression coefficients, male, age of ≥36 years, a spleen below a costal margin of ≥7 cm, and an HGB level of <100 g/L were given 1 point to form a predictive system. All patients were divided into three risk subgroups, and the incidence of severe cytopenia significantly differed among the three groups（*P* < 0.001）. Grade ≥3 neutrophils and/or thrombocytopenia for >2 weeks was significantly associated with lower cumulative incidences of complete cytogenetic response（CCyR, *HR*＝0.5, 95％ *CI* 0.3–0.7, *P*<0.001）and major molecular response（MMR, *HR*＝0.4, 95％ *CI* 0.3–0.8, *P*＝0.004）and was not significantly associated with failure, progression, and survival.

**Conclusion:**

Male, advanced age, a large spleen, and a low HGB level were significantly associated with severe cytopenia. The four covariates were used to establish a prediction model, in which the incidence of severe cytopenia among different risk groups was significantly different. Severe cytopenia for >2 weeks was a negative factor for responses but not for outcomes.

2000年，伊马替尼极大地改善了慢性髓性白血病慢性期（CML-CP）患者的生存[1]。而二代酪氨酸激酶抑制剂（TKI）如尼洛替尼和达沙替尼的出现使患者更快达到更深的治疗反应，并克服少部分伊马替尼耐药，已成为一部分患者的一线治疗选择[2]–[3]。血细胞减少是TKI治疗的常见不良反应之一，是CML患者停药和减量的重要指征[4]。研究显示，应用二代TKI的患者血细胞减少的发生率与应用伊马替尼的患者相当，甚至更高[5]–[11]。目前，对于一线治疗使用二代TKI患者的血细胞减少的相关因素及临床意义报道较少。为此我们回顾性分析2008年起在北京大学人民医院确诊为CML-CP并使用二代TKI药物作为一线治疗的成年患者，分析严重血细胞减少的相关因素及其对于治疗反应、结局的影响。

## 病例与方法

一、病例

纳入2008年9月至2021年11月间在北京大学人民医院确诊为CML-CP，并使用二代TKI作为一线治疗的成年连续病例。排除初诊到治疗超过6个月、不规律随访以及初始治疗未使用标准剂量（如尼洛替尼300 mg每日2次或达沙替尼100 mg每日1次）者。从病历中收集患者信息，包括性别、确诊时年龄、脾脏大小、外周血HGB浓度、WBC、PLT、原始细胞比例、嗜碱性粒细胞比例、合并症、TKI种类及剂量、≥ 3级中性粒细胞和（或）血小板减少发生情况，以及治疗反应和结局，根据患者信息计算ELTS评分。

二、诊断分期

CML的诊断、分期参照ELN指南[3],[12]–[13]。

三、治疗

TKI的选择和剂量调整参照ELN指南[3],[12]–[13]。

根据患者的年龄、疾病危险度、合并症、合并用药以及经济情况等，由患者和医师共同决定一线TKI药物，初始剂量为尼洛替尼300 mg每日2次或达沙替尼100 mg每日1次。当发生≥3级的中性粒细胞或血小板减少时停药，直至中性粒细胞计数（ANC）>1.0×10^9^/L，PLT>50×10^9^/L时恢复用药。若停药时间超过2周，恢复用药时剂量减少1/4～1/3。

四、监测及随访

监测方法和频率参照ELN指南[3]–[4],[12]–[13]。

血液学：治疗前3个月每1～2周进行血细胞分类计数，治疗第3～6个月，每1～2个月检测1次；之后每3个月检测1次。细胞遗传学：采用骨髓进行染色体核型分析，每3～6个月评估1次直至获得完全细胞遗传学反应（CCyR）。分子学反应：仅评估b2a2/b2a3转录本患者，采用外周血标本，利用实时定量PCR（qRT-PCR）监测BCR-ABL水平，每3个月监测1次直至达到完全分子学反应（MMR），之后每3～6个月监测1次。采用门诊、查阅病历或电话联系方式进行随访。随访截止时间为2022年4月。

五、评估指标

1. 血细胞减少：因为HGB不作为药物剂量调整的指标，故本研究仅关注中性粒细胞减少及血小板减少。根据NCI-CTC定义血细胞减少[4]。3级中性粒细胞和血小板减少分别定义为ANC<1.0×10^9^/L，PLT<50×10^9^/L。4级中性粒细胞和血小板减少分别定义为ANC<0.5×10^9^/L，PLT<25×10^9^/L。

2. 治疗反应：根据2020年ELN指南[3],[12]–[13]定义完全血液学反应（CHR）、CCyR、MMR、分子学反应4（MR4）和分子学反应4.5（MR4.5）。

3. 结局：无失败生存（FFS）期定义为从开始TKI治疗至治疗失败或末次随访的时间；无进展生存（PFS）期定义为从开始TKI治疗直至进展到加速期/急变期、疾病进展所致的死亡或末次随访的时间；总生存（OS）期定义为从开始TKI治疗至死亡或末次随访的时间；所有结局均以造血干细胞移植作为删失事件。

六、统计学处理

1. 描述性统计：对患者信息进行描述性统计，分类变量用频数和频率进行描述，连续变量用中位数和范围进行描述。

2. 分析≥3级中性粒细胞和（或）血小板减少的相关因素：采用受试者工作特征曲线（ROC）确定连续变量对于发生严重血细胞减少的最佳截断值，若截断值无法确定，则用中位数或公认值代替。应用单、多因素二元Logistic回归模型分析与≥3级中性粒细胞和（或）血小板减少显著相关的变量，单因素分析中*P*<0.2的变量纳入多因素分析。依据多因素分析中显著变量的回归系数赋分建立预测模型[14]。

3. 分析≥3级中性粒细胞和（或）血小板减少对治疗反应和结局的影响：采用X-tile软件确定连续变量对于治疗反应、结局的最佳截断值，若截断值无法确定，则用中位数或公认值代替。对于累积治疗反应（CCyR、MMR、MR4、MR4.5）获得率采用竞争风险模型，应用Fine-gray检验进行组间比较，以移植和死亡作为竞争风险事件；对于结局（FFS、PFS）采用Kaplan-Meier生存曲线分析，应用Log-rank检验进行组间比较。由于总人群中死亡人数较少，本研究不评估血细胞减少对总生存（OS）的影响。单因素分析中*P*<0.2的变量被纳入Cox回顾模型进行多因素分析。*P*<0.05时认为差异有统计学意义。本研究采用SPSS 22.0、R 4.0.2、X-tile软件进行数据分析。

## 结果

一、患者特征

共收集267例一线二代TKI治疗的CML-CP连续患者，中位年龄36（18～73）岁，男性156例（58.4％）。ELTS评分低、中和高危分别为149例（55.8％）、67例（25.1％）和36例（13.5％）。有合并症者84例（31.5％）。诊断到治疗时间≥2个月者30例（11.2％）。服用尼洛替尼300 mg每日2次者239例（89.5％），服用达沙替尼100 mg每日1次者28例（10.5％）（表1）。各变量之间不存在共线性。

**表1:** 267例接受一线二代TKI治疗的慢性髓性白血病慢性期患者基线特征

变量	数值
年龄[岁，*M*（范围）]	36（18~73）
男性[例（%）]	156（58.4）
ELTS评分[例（%）]	
低危	149（55.8）
中危	67（25.1）
高危	36（13.5）
未知	15（5.6）
脾脏肋缘下[cm，*M*（范围）]	4.2（0~26.0）
WBC[×10^9^/L，*M*（范围）]	156.2（7.8~754.7）
HGB[g/L，*M*（范围）]	111（64~167）
PLT[×10^9^/L，*M*（范围）]	441（28~2 887）
外周血原始细胞[%，*M*（范围）]	1（0~13）
外周血嗜碱性粒细胞[%，*M*（范围）]	5（0~21）
有合并症[例（%）]	84（31.5）
诊断到治疗时间≥2个月[例（%）]	30（11.2）
TKI[例（%）]	
尼洛替尼600 mg/d	239（89.5）
达沙替尼100 mg/d	28（10.5）

**注** ELTS评分：EUTOS长期生存评分；TKI：酪氨酸激酶抑制剂

中位TKI治疗时间43（3～161）个月，截至随访期末，251例（94.0％）患者在TKI治疗中，其中217例（81.3％）仍服用二代TKI治疗，包括尼洛替尼177例（66.3％），达沙替尼40例（15.0％）；12例（4.5％）因经济原因、8例（3.0％）因不耐受以及5例（1.9％）因耐药换用伊马替尼治疗；9例（3.4％）因耐药换用三代TKI治疗。4例（1.5％）为追求无治疗缓解（TFR）、3例（1.1％）因不耐受、3例（1.1％）因备孕、1例（0.4％）因经济原因以及1例（0.4％）因合并症停药。3例（1.1％）在疾病进展后以及1例（0.4％）为追求更好疗效接受异基因造血干细胞移植。

二、发生严重血细胞减少的相关因素

全部267例一线二代TKI治疗的患者中，43例（16.1％）发生了≥3级中性粒细胞和（或）血小板减少，其中12例（4.5％）发生中性粒细胞减少，41例（15.4％）发生血小板减少，10例（3.7％）二者均发生。中位发生时间为一线治疗开始后1.0（0.1～3.0）个月，中位持续时间为1.0（0.1～24.6）个月，其中7例（16.3％）持续时间≤2周，以初始剂量恢复用药；36例（83.7％）持续时间>2周，以初始剂量的2/3～3/4恢复用药，其中14例（38.9％）在后续治疗中逐渐恢复原剂量，11例（30.6％）维持低剂量用药，8例（22.2％）因减量后仍反复出现血细胞减少而换药，2例（5.6％）因反复出现血细胞减少而间断停药,1例（2.8％）因反复出现血细胞减少而进行移植。

采用ROC曲线确定初诊时脾脏肋缘下大小、WBC、HGB和外周血原始细胞比例对于≥3级中性粒细胞和（或）血小板减少的最佳截断值，取整后分别为：脾脏肋缘下7.0 cm，WBC 130×10^9^/L，HGB 100 g/L以及外周血原始细胞比例7％。年龄、PLT、外周血嗜碱性粒细胞比例以及诊断到治疗时间无法得出有意义的截断值，故诊断到治疗时间分为<2个月和≥2个月两组，年龄、PLT和外周血嗜碱性粒细胞比例采用中位数为截断值，分别为36岁、440×10^9^/L和5％。

采用二元Logistic模型分析≥3级中性粒细胞和（或）血小板减少的相关因素，包括性别、诊断时年龄、脾脏肋缘下大小、HGB、WBC、PLT、外周血原始细胞比例、外周血嗜碱性粒细胞比例、合并症、诊断到治疗时间以及TKI种类。单因素分析结果见表2，多因素分析结果显示：男性、初诊时年龄≥36岁、脾脏肋缘下≥7 cm以及初诊时HGB<100 g/L与≥3级中性粒细胞和（或）血小板减少的发生显著相关（表2）。

**表2:** 二代酪氨酸激酶抑制剂一线治疗的慢性髓性白血病慢性期患者发生≥3级中性粒细胞和（或）血小板减少的影响因素分析

变量	单因素分析		多因素分析	
	*P*值	*OR*（95%*CI*）	*P*值	*OR*（95%CI）
性别（女/男）	0.022	0.4（0.2~0.9）	0.018	0.4（0.1~0.8）
年龄（≥36岁/<36岁）	0.083	1.8（0.9~3.5）	0.005	3.2（1.4~7.2）
脾脏肋缘下	<0.001		0.060	
<7.0 cm（参照）		1		1
≥7.0 cm	<0.001	4.6（2~2.9.7）	0.020	2.8（1.2~6.6）
未知	0.072	3.2（0.9~11.0）	0.190	2.6（0.6~10.6）
WBC	0.001			
<130×10^9^/L（参照）		1		
≥130×10^9^/L	<0.001	5.7（2.3~14.0）		
未知	1.000	0		
HGB	<0.001		0.007	
<100 g/L（参照）		1		1
≥100 g/L	<0.001	0.2（0.1~0.5）	0.012	0.3（0.1~0.8）
未知	0.156	3.8（0.6~24.3）	0.189	5.0（0.5~56.2）
PLT	0.586			
<440×10^9^/L（参照）		1		
≥440×10^9^/L	0.314	0.7（0.4~1.4）		
未知	0.918	1.1（0.1~10.5）		
外周血原始细胞	0.002			
<7%（参照）		1		
≥7%	<0.001	3.8（1.8~7.9）		
未知	0.715	1.3（0.3~6.4）		
外周血嗜碱性粒细胞	0.666			
<5%（参照）		1		
≥5%	0.785	0.9（0.5~1.8）		
未知	0.447	1.7（0.4~6.9）		
合并症（有/无）	0.878	0.9（0.5~1.9）		
诊断到治疗时间(≥2个月/<2个月）	0.157	0.3（0.8~1.5）		
治疗（达沙替尼100 mg/d/尼洛替尼600 mg/d）	0.061	2.4（1.0~5.8）		

根据各变量回归系数赋分，建立≥3级中性粒细胞和（或）血小板减少的预测模型：女性、初诊年龄<36岁，脾脏肋缘下<7 cm以及HGB≥100 g/L赋0分；男性、初诊年龄≥36岁，脾脏肋缘下≥7 cm以及HGB<100 g/L赋1分。合并血细胞减少发生率差异无统计学意义的分数组，最终将信息完整的243例患者分为3个亚组：低危组（0～1分，105例，43.2％），中危组（2分，68例，28.0％）以及高危组（3～4分，70例，28.8％）。各组血细胞减少发生率分别为2.9％、14.7％和32.9％（*P*≤0.001），以低危组为参考，中危和高危组发生血细胞减少的*OR*值分别为5.9（95％*CI* 1.6～22.2）和16.6（95％*CI* 4.8～58.2），各危险组患者血液学不良反应发生时间和持续时间差异无统计学意义。

三、发生严重血细胞减少对治疗反应和结局的影响

1. 发生严重血细胞减少对治疗反应的影响：全部267例患者中，259例（97.0％）获得CHR。中位TKI治疗时间43（3～161）个月，241例（90.3％）获得CCyR，261例（97.8％）b2a2/b2a3转录本患者评估了分子学反应，其中209例（80.1％）获得MMR，123例（47.1％）获得MR4，86例（33.0％）获得MR4.5。6年累积CCyR、MMR、MR4和MR4.5获得率分别为95.0％（95％*CI* 91.9％～98.1％）、89.8％（95％*CI* 85.1％～94.5％）、67.0％（95％*CI* 58.2％～75.8％）和43.4％（95％*CI* 35.4％～51.4％）。

采用X-tile软件确定初诊时WBC和HGB对于分子学反应的最佳截断值，取整后分别为：WBC 30.0×10^9^/L和100.0×10^9^/L，HGB 120 g/L。外周血嗜碱性粒细胞比例无法得出有意义的截断值，采用中位数5％。

采用竞争风险模型分析影响治疗反应的因素，包括性别、诊断时年龄、ELTS评分、HGB、WBC、外周血嗜碱性粒细胞比例、合并症、TKI种类以及发生严重血细胞减少的情况。多因素分析显示：发生≥3级中性粒细胞和（或）血小板减少>2周与较低的CCyR以及MMR获得率显著相关（表3，图1）。此外，ELTS中高危与较低的CCyR、MMR以及MR4获得率显著相关；初诊时高WBC（≥30×10^9^/L）与较低的MMR、MR4以及MR4.5获得率显著相关；男性与较低的MMR获得率显著相关。

**表3:** 二代酪氨酸激酶抑制剂一线治疗的慢性髓性白血病慢性期患者治疗反应和结局的多因素分析结果

变量	CCyR		MMR		MR4	
	*HR*（95%*CI*）	*P*值	*HR*（95%*CI*）	*P*值	*HR*（95%*CI*）	*P*值
性别（女/男）			1.4（1.0~1.8）	0.033		
ELTS评分		0.001		0.002		0.024
低危（参照）	1		1		1	
中危	0.8（0.6~1.0）	0.032	0.8（0.6~1.1）	0.240	1.3（0.8~2.0）	0.250
高危	0.4（0.3~0.7）	<0.001	0.4（0.2~0.6）	<0.001	0.4（0.2~0.9）	0.034
未知	0.8（0.5~1.2）	0.340	0.7（0.5~1.2）	0.210	0.5（0.2~1.1）	0.068
WBC（×10^9^/L）				0.031		0.010
<30.0（参照）			1		1	
30.0~100.0			0.6（0.4~0.9）	0.027	0.5（0.3~0.9）	0.032
≥100.0			0.6（0.4~0.9）	0.007	0.4（0.2~0.7）	0.002
未知			1.2（0.4~3.5）	0.750	-	<0.001
严重血细胞减少^a^		<0.001		0.002		
未发生（参照）	1		1			
发生≤2周	0.9（0.6~1.3）	0.560	0.9（0.4~1.9）	0.700		
发生>2周	0.5（0.3~0.7）	<0.001	0.4（0.3~0.8）	0.004		

变量	MR4.5		FFS		PFS	
	*HR*（95%*CI*）	*P*值	*HR*（95%*CI*）	*P*值	*HR*（95%*CI*）	*P*值
性别（女/男）						
ELTS				<0.001		<0.001
低危（参照）			1		1	
中危			1.3（0.7~2.6）	0.420	2.1（0.8~6.1）	0.154
高危			5.5（3.0~10.1）	<0.001	8.5（3.3~21.5）	<0.001
未知			0.9（0.2~3.7）	0.386	1.3（0.2~10.6）	0.804
WBC（×10^9^/L）		<0.001				
<30.0（参照）	1					
30.0~100.0	0.4（0.2~0.8）	0.007				
≥100.0	0.3（0.1~0.5）	<0.001				
未知	-	<0.001				
严重血细胞减少^a^						
未发生（参照）						
发生≤2周						
发生>2周						

**注** ^a^指≥3级中性粒细胞和（或）血小板减少；ELTS评分：EUTOS；长期生存评分；CCyR：完全细胞遗传学反应；MMR：完全分子学反应；MR4：分子学反应4；MR4.5：分子学反应4.5；FFS：无失败生存；PFS：无进展生存

**图1:**
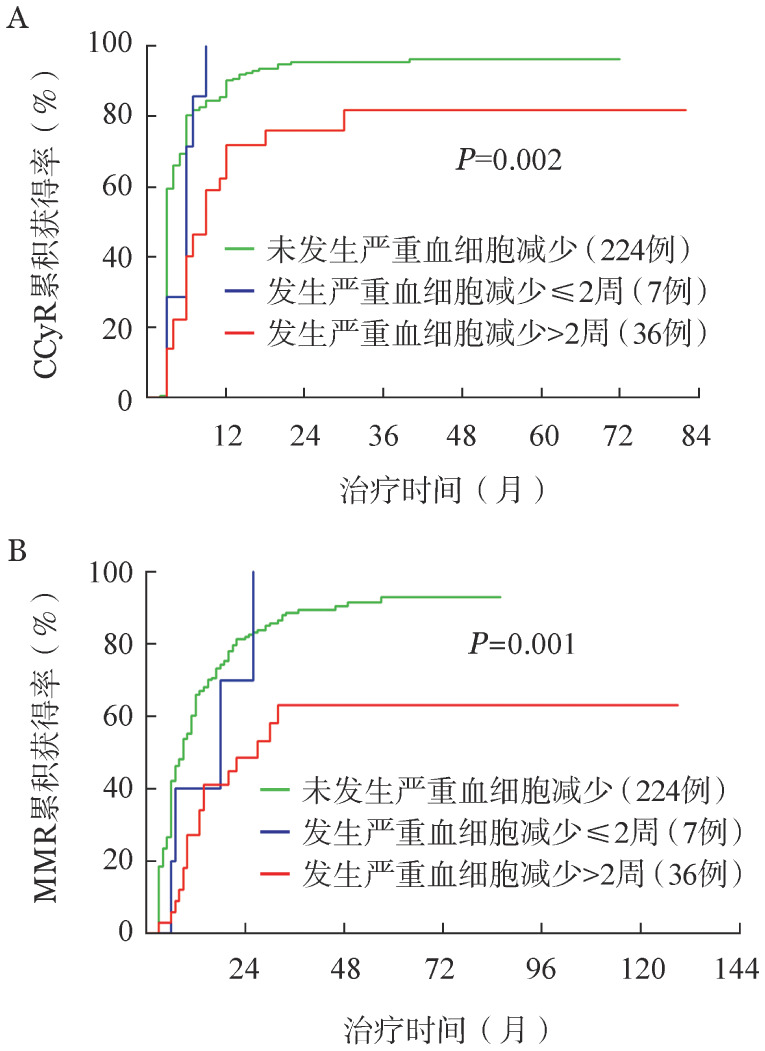
≥3级中性粒细胞和（或）血小板减少对二代TKI一线治疗的CML患者CCyR（A）以及MMR（B）的影响 TKI：酪氨酸激酶抑制剂；CML：慢性髓性白血病；CCyR：完全细胞遗传学反应；MMR：主要分子学反应

2. 发生严重血细胞减少对结局的影响：全部患者中58例（21.7％）发生治疗失败，27例（10.1％）进展为加速期或急变期，6例（2.2％）死亡，4例（1.5％）在疾病进展后、1例（0.4％）因治疗中反复出现血小板减少而接受移植。6年FFS率、PFS率和OS率分别为76.6％（95％ *CI* 71.4％～81.8％）、87.9％（95％ *CI* 83.4％～92.4％）和95.7％（95％ *CI* 92.0％～99.4％）。

采用X-tile软件确定初诊时WBC、HGB和外周血嗜碱性粒细胞比例对于FFS和PFS的最佳截断值，取整后分别为WBC 70.0×10^9^/L、HGB 120 g/L、外周血嗜碱性粒细胞比例7.5％。

采用Cox模型分析影响结局的因素，包括性别、诊断时年龄、ELTS评分、HGB、WBC、外周血嗜碱性粒细胞比例、合并症、TKI种类以及发生严重血细胞减少的情况。多因素分析显示：发生≥3级中性粒细胞和（或）血小板减少>2周与FFS以及PFS均无显著相关。ELTS中高危与较差的FFS和PFS显著相关（表3）。

## 讨论

本研究回顾性分析了本所一线使用二代TKI的初诊成年CML-CP病例，结果显示：16.1％的患者在一线治疗开始后1.0（0.1～3.0）个月发生了≥3级血液学不良反应。男性、初诊年龄≥36岁、脾脏肋下≥7 cm以及HGB<100 g/L与≥3级中性粒细胞和（或）血小板减少显著相关。联合患者性别、初诊年龄、脾脏肋缘下大小以及HGB浓度建立预测模型，不同危险组患者严重血细胞减少的发生率差异有统计学意义。≥3级中性粒细胞和（或）血小板减少>2周与较低的CCyR和MMR获得率显著相关，与二代TKI治疗失败、疾病进展以及生存期无关。

国际上多项研究报道，一线治疗中≥3级中性粒细胞减少以及≥3级血小板减少的发生率在尼洛替尼300 mg每日2次时分别为12％～14.8％和10％～15.0％[7],[10]–[11]，在达沙替尼100 mg每日1次时分别为19.4％～21.0％和17.4％～19.0％[5]–[6],[8]–[9]。本研究中，患者主要使用尼洛替尼，≥3级血小板减少发生率为15.4％，与国外报道相似；≥3级中性粒细胞减少发生率为4.5％，低于国外报道，也低于ENESTchina研究报道的21.1％[11]。ENESTchina研究显示，对于服用尼洛替尼的中国CML-CP患者，血小板减少是导致停药或剂量调整最常见的药物不良反应[11]。故推测可能由于治疗过程中血小板减少的发生早于中性粒细胞减少，患者的停药降低了≥3级中性粒细胞减少的发生。

ELN推荐指出，血细胞减少最常发生在治疗开始的1周或1个月内，治疗后第4～6周最严重，随着治疗进行而逐渐缓解[4]，与本研究发现一致。本中心既往研究显示，一线伊马替尼治疗的患者≥3级中性粒细胞和（或）血小板减少中位发生时间为用药后1.0（0.1～7.0）个月，持续时间为0.6（0.3～6.5）个月[15]。本研究中一线二代TKI治疗的患者≥3级中性粒细胞和（或）血小板减少持续时间为1.0（0.1～24.6）个月，提示一线二代TKI治疗患者血液学不良反应持续时间较一线应用伊马替尼患者更长。

既往针对严重血液学不良反应影响因素的研究多以伊马替尼治疗的患者作为研究对象[15]–[20]。McLigeyo等[18]一项纳入201例伊马替尼治疗病例的研究显示，初诊时HGB<79 g/L、ANC<1.5×10^9^/L、PLT<150×10^9^/L或>450×10^9^/L与血细胞减少的发生显著相关。Cortes等[16]一项纳入338例伊马替尼治疗病例的研究显示，初诊时HGB<120 g/L、≥ 60岁、女性、初始高剂量、Sokal中危和高危与治疗过程中发生贫血显著相关。本中心既往研究显示，以一线伊马替尼治疗患者为主要研究对象时，女性、诊断时WBC ≥100×10^9^/L和CP-Sokal高危、原始细胞增多型加速期是严重血细胞减少发生的高危因素[15]。本研究发现，对于一线二代TKI治疗患者，初诊时HGB、Sokal评分中的年龄和脾脏肋缘下大小与≥3级中性粒细胞和（或）血小板减少的发生显著相关，可能与这部分患者疾病危险度更高、体内白血病负荷较高以及正常造血克隆较少相关。值得注意的是，本研究中男性是≥3级中性粒细胞和（或）血小板减少发生的危险因素，与既往研究有所出入，需要进一步研究。此外，本研究中单因素分析显示，相比于尼洛替尼，使用达沙替尼更容易发生严重血细胞减少，与既往研究结果相符。

既往Sneed等[21]纳入143例伊马替尼治疗CML-CP病例的研究显示，≥3级血细胞减少与主要细胞遗传学反应以及CCyR显著相关，当血细胞减少持续>2周时影响更显著。本研究显示，对于一线应用二代TKI的患者，发生≥3级中性粒细胞和（或）血小板减少>2周不仅与较低的CCyR获得率相关，还与较低的MMR获得率显著相关。分析其原因，若≥3级血细胞减少发生>2周，患者将被迫以低剂量恢复用药，可能影响治疗反应获得的速度和深度。另外，发生严重血细胞减少的患者可能白血病负荷较高、正常造血克隆较少并且疾病危险度较高，导致治疗反应的获得率较低。值得注意的是，相较于既往研究中的一线应用伊马替尼患者，本研究中一线应用二代TKI患者血液学不良反应持续时间更长，更有可能影响患者治疗反应的获得，需要更多关注。

本研究存在以下局限性：①回顾性研究；②单中心研究，开展于大型三级甲等教学医院，可能存在选择偏倚；③未进一步收集依从性数据。

总之，本研究显示，男性、年龄≥36岁、脾脏肋缘下≥7 cm以及HGB<100 g/L与初发CML-CP患者服用二代TKI期间发生严重血细胞减少显著相关，联合四者建立预测模型，不同危险组患者严重血细胞减少的发生率有显著差异。严重血细胞减少持续>2周是细胞遗传学和分子学反应的不利因素。
